# Transarterial chemoembolization with molecular targeted therapies plus camrelizumab for recurrent hepatocellular carcinoma

**DOI:** 10.1186/s12885-024-12144-6

**Published:** 2024-03-27

**Authors:** Changlong Hou, Baizhu Xiong, Lei Zhou, Yipeng Fei, Changgao Shi, Xianhai Zhu, Tao Xie, Yulin Wu

**Affiliations:** 1 Department of Intervention, Division of Life Sciences and Medicine, The First Affiliated Hospital of USTC, University of Science and Technology of China, 107# Huanhu East Road, Shushan District, 230031 Hefei, Anhui People’s Republic of China; 2 Graduate School of Bengbu Medical College, Bengbu, Anhui China

**Keywords:** Hepatocellular carcinoma, Transarterial chemoembolization, Targeted therapy, Immune checkpoint inhibitors, Interventional treatment

## Abstract

**Background:**

The safety and efficacy of transarterial chemoembolization plus molecular targeted therapy (MTT) combined with immune checkpoint inhibitors (ICIs) in primary liver cancer have been demonstrated. However, the evidence for TACE plus MTT combined with ICIs in the treatment of recurrent hepatocellular carcinoma (RHCC) is limited. Given the excellent performance of this combination regimen in primary liver cancer, it is necessary to evaluate the efficacy of TACE plus MTT combined with ICIs in RHCC.

**Methods:**

A total of 88 patients with RHCC treated with TACE plus MTT combined with camrelizumab (TACE-TC group, *n* = 46) or TACE plus MTT (TACE-T group, *n* = 42) were retrospectively collected and analyzed. In this study, we evaluated the effectiveness and safety of combination therapy for patients with RHCC by analyzing tumor response, progression-free survival (PFS), overall survival (OS), laboratory biochemical indices, and adverse events (AEs).

**Results:**

TACE-TC was superior to TACE-T in PFS (14.0 vs. 8.9 months, *p* = 0.034) and OS (31.1 vs. 20.2 months, *p* = 0.009). Moreover, TACE-TC achieved more preferable benefits with respect to disease control rate (89.1% vs. 71.4%, *p* = 0.036) and objective response rate (47.8% vs. 26.2%, *p* = 0.036) compared with TACE-T in patients with RHCC. Compared with the TACE-T group, the AFP level in the TACE-TC group decreased more significantly after 3 months of treatment. Multivariate analysis showed that treatment option was a significant predictor of OS and PFS, while the portal vein tumor thrombus and interval of recurrence from initial treatment were another prognostic factor of PFS. There was no significant difference between the TACE-TC and TACE-T groups for Grade 3–4 adverse events.

**Conclusions:**

A combination therapy of TACE, MTT, and camrelizumab significantly improved tumor response and prolonged survival duration, showing a better survival prognosis for RHCC patients.

## Introduction

Hepatocellular carcinoma (HCC) is one of the most common malignant tumors worldwide [1]. Liver resection, as the first-line treatment for patients with early-stage HCC, has a recurrence rate as high as 60–80% within 5 years after surgery [2]. Liver resection is a viable option for treating recurrent hepatocellular carcinoma (RHCC), but it should be noted that this differs from primary HCC due to limitations in residual liver function or advanced tumor stage. Many RHCC patients may not be eligible for a second surgery. Local ablative therapy is a cost-effective treatment option with comparable survival rates to resection [3]. However, eligibility for ablation depends on factors such as tumor size, location, and the ability to achieve sufficient ablation margins [4]. For HCC larger than 3 cm and those situated near critical structures like large vessels, diaphragm, heart, or central bile ducts, alternative locoregional modalities may be more suitable [5].

Transarterial chemoembolization (TACE) is a well-tolerated and limited hepatotoxic technique that serves as an alternative to liver resection. It combines targeted chemotherapy with arterial embolization-induced ischemic necrosis and can be used for any stage of HCC. The embolization effect of TACE can induce a localized hypoxic environment, thereby promoting the expression of vascular endothelial growth factor (VEGF) and stimulating tumor angiogenesis. This process may potentially result in a subsequent tumor recurrence or metastasis [6, 7]. The effect of TACE is closely related to the patient’s prognosis, especially long-term survival time [8]. With sorafenib and lenvatinib, which are multikinase inhibitors with antiproliferative and antiangiogenic activities recommended as first-line treatment for advanced hepatocellular carcinoma [9, 10], the combination of TACE and molecular targeted therapies (MTT) is thought to be effective in reversing TACE hypoxia-induced angiogenesis and improving the outcome of treatment [11]. Several clinical studies have shown that the efficacy of TACE combined with MTT in the treatment of advanced HCC is better than that of TACE or MTT alone [12–14]. The efficacy analysis of TACE combined with MTT in the treatment of RHCC has also been reported [15, 16], which is also superior to TACE or MTT alone.

On the other hand, the ischemia and cytotoxic damage that TACE causes to the tumor may make it easier to prime de novo T-cell responses against tumor-associated antigens, thus enabling an increased activity of PD-1/PD-L1 inhibitors [17, 18]. And the immunosuppressive tumor microenvironment can be transformed into an immunostimulatory milieu by VEGF inhibitors [19]. Under these circumstances, administering PD-1/PD-L1 antibodies boosts T cells’ anticancer activity. Therefore, TACE and MTT combined with PD-1 inhibitors are considered to be one of the effective treatment strategies for advanced HCC [20, 21]. A recent study [22] showed that the survival benefit of TACE plus lenvatinib combined with PD-1 inhibitors in the treatment of RHCC is better than that of TACE combined with lenvatinib. This suggests that the triple regimen may be equally effective in RHCC.

Camrelizumab, a PD-1 monoclonal antibody independently developed in China, has been approved as a second-line treatment for unresectable HCC [23]. It exhibits high affinity while possessing good safety profile [24, 25]. Given the possible tumor heterogeneity between recurrent HCC and primary HCC, it has potential clinical implications [26]. At present, no studies have investigated whether TACE combined with MTT and then combined with camrelizumab can improve the efficacy of TACE combined with MTT in the treatment of RHCC. Therefore, this retrospective clinical study was conducted to evaluate the efficacy and safety of TACE plus MTT combined with camrelizumab in treating patients with RHCC.

## Materials and methods

### Study Population

Data of consecutive patients with RHCC who received TACE plus MTT combined with camrelizumab (TACE-TC) or TACE plus MTT (TACE-T) at our institution between January 2016 and June 2022 were retrospectively analyzed. RHCC is defined as the recurrence of HCC after radical liver resection. The main inclusion criteria are: (1) all patients were pathologically confirmed to have HCC; (2) patients with first recurrence after radical liver resection; (3) according to the American Association for the Study of Liver Diseases Practice Guideline for Management of HCC [27], RHCC is diagnosed using imaging investigations (triphasic CT and/or MRI) revealing both early enhancement and delayed decreasing enhancement; (4) Child-Pugh A or Child‐Pugh B; (5) The tumor burden must meet at least one of the three criteria: invasion of large blood vessels, extrahepatic metastasis, or tumor diameter ≥ 5 cm. Following are the exclusion criteria: (1) Previously received systemic therapy (including molecular targeted therapy and immunotherapy); (2) Patients with diffuse intrahepatic recurrence; (3) Treatment was discontinued due to serious adverse events or other reasons.

### Treatment protocol

The operation procedure of TACE is as follows: the modified Seldinger technique was used to puncture the femoral artery and insert the catheter sheath, and then the catheter was inserted into the tumor-supplying artery. Inject an appropriate amount of emulsifier made of epirubicin (10–40 mg) and lipiodol embolic (10–20 ml, Jiangsu Hengrui Medicine Co, China) for tumor embolization. Finally use gelatin sponge or polyvinyl alcohol pellets (300–500 μm, Beijing Fu’aile Technology Development Co, China) to strengthen the embolization in an appropriate amount until the tumor staining by DSA disappeared.

MTT was initiated within 7 days of the first TACE. Including lenvatinib (8 mg or 12 mg), sorafenib (800 mg), or apatinib (400 mg), orally daily. Patients in the TACE-TC group were additionally combined with camrelizumab, which was administered intravenously at a standard dose (200 mg/3 weeks). For patients with extrahepatic metastasis, the multidisciplinary team (MDT) will recommend the TACE + MTT + ICI first. For patients with larger intrahepatic tumors, TACE + MTT is recommended first. Then, patients are informed of the advantages and disadvantages of all treatment options, as well as potential treatment results, related adverse reactions, and treatment costs before making a final decision. Subsequent treatment options include targeted drugs, PD-1 inhibitors, radiotherapy, or best supportive care.

### Follow-up and efficacy assessment

All patients were followed up every 6–8 weeks after initial treatment, including routine blood tests, blood biochemical tests, contrast-enhanced abdominal CT or MRI, and other sites if indicated. Repeat TACE depends on whether there is residual arterial phase enhancement in the target lesion. Tumor response was evaluated according to mRECIST criteria [28], which were mainly classified into complete response (CR), partial response (PR), stable disease (SD), or progressive disease (PD). The objective response rate (ORR) was defined as the percentage of patients in CR and PR. The disease control rate (DCR) was defined as the percentage of patients in CR, PR, and SD. Adverse events (AEs) were graded according to the CTCAE version 5.0 criteria. The primary endpoint of this study was OS, which is the duration from relapse diagnosis to either death or the end of the follow-up period. The secondary endpoints were PFS, ORR, and DCR. PFS was defined as the time between the first TACE after relapse diagnosis and the date of assessment of tumor progression or patient death. We also conducted subgroup analysis based on microvascular invasion status and interval of recurrence from initial surgical treatment.

Treatment with TACE-TC or TACE-T was stopped during follow-up in cases of PD. Following discussions with our multidisciplinary team and the patient’s requests, the best course of treatment, which may include radiation, PD-1 inhibitors, targeted medications, or optimal supportive care, was decided.

### Statistical analysis

All the data were statistically carried out using Medcalc, SPSS (version 20.0), and R (version 4.1.3) software. For the baseline characteristics, continuous variables are described as the mean ± standard deviation or median ± interquartile range and using independent sample t-test or Mann-Whitney U test to compare two groups. Categorical variables are expressed as the number of patients, and the chi-square test or Fisher’s exact probability was used for comparison between the two groups. The univariable Cox proportional hazards model was used to analyze each variable. Variables with a two-sided *p*-value of < 0.1 were then included in the multivariable analysis. A Cox hazard regression model was employed to identify their value as independent predictors of overall survival and progression-free survival. Kaplan-Meier curves were plotted for survival analysis. Statistically significant differences were defined as *p*-value of < 0.05.

## Results

### Patient demographics

**Fig. 1:**
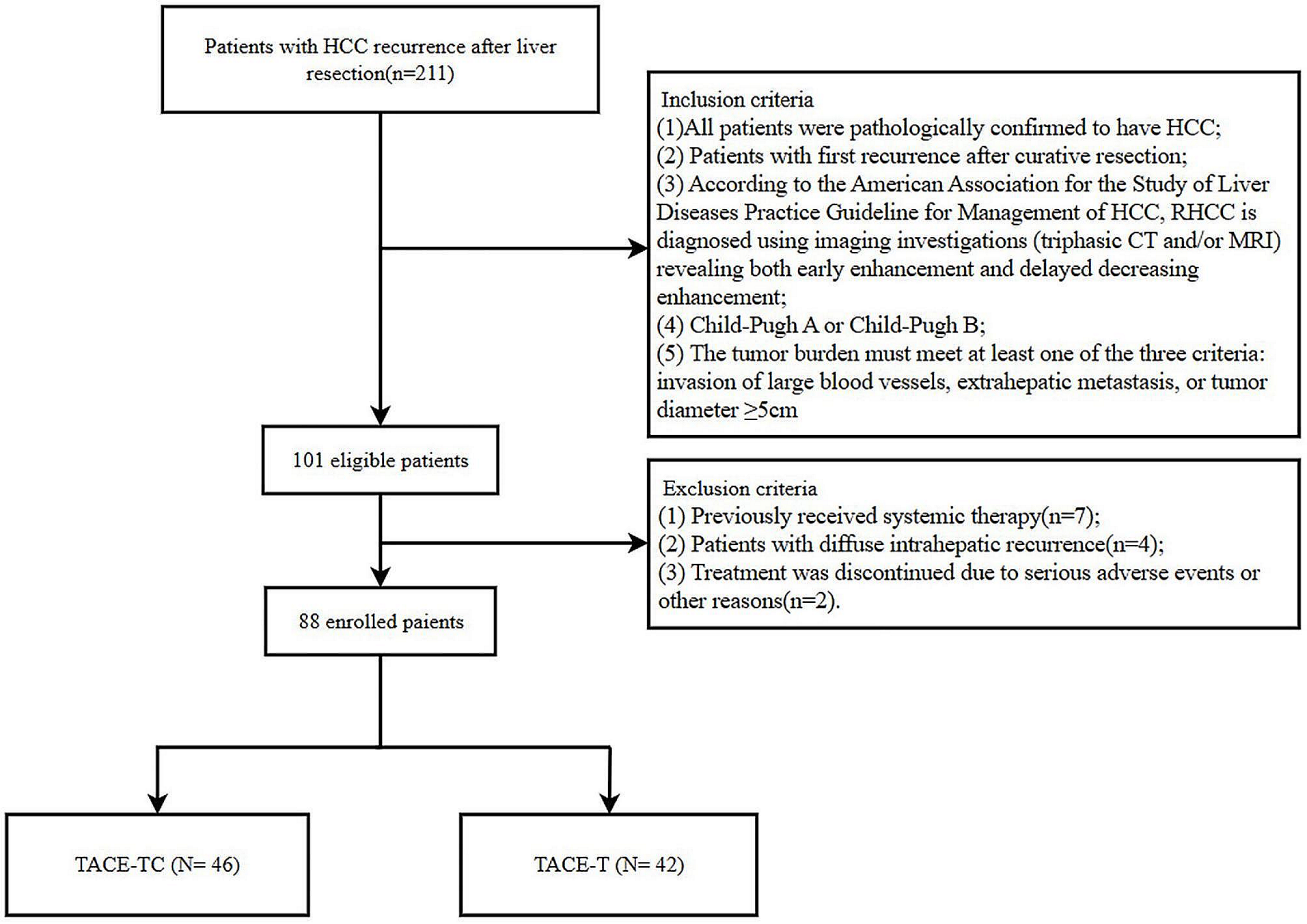
Flow diagram of patient enrollment. TACE, Transarterial chemoembolization; TACE-TC, TACE plus molecular targeted therapies combined with camrelizumab; TACE-T, TACE plus molecular targeted therapies

This study finally included 88 RHCC patients (Fig. 1). The baseline characteristics table (Table 1) provides important insights regarding the study participants. Regarding age, the overall group was 55 ± 11 years, with slightly lower means observed in the TACE-T group (53 ± 9 years) and slightly higher means in the TACE-TC group (57 ± 12 years). The majority of participants were male, with percentages of 90.9%, 85.7%, and 95.7% for the overall, TACE-T, and TACE-TC groups, respectively. The Child-Pugh classification showed that the majority of participants were classified as grade A (89.8% overall, 90.5% TACE-T, and 89.1% TACE-TC). In the TACE-TC group, patients received the following classes of tyrosine kinase inhibitors: apatinib in 30 cases (65.2%), average duration 13.14 months, sorafenib in 8 cases (17.4%), average duration 17.48 months, and lenvatinib in 8 cases (17.4%), average duration 19.70 months. There was no significant difference in baseline characteristics between the TACE-TC group and the TACE-T group.

**Table 1:** Patient demographics and baseline characteristics

Characteristic	Group			P
	Overall (*N* = 88)	TACE-T(*n* = 42)	TACE-TC(*n* = 46)	
**Age (year)**	55.0 ± 11.0	53.0 ± 9.0	57.0 ± 12.0	0.126
**Sex**				0.145
Male	80 (90.9%)	36 (85.7%)	44 (95.7%)	
Female	8 (9.1%)	6 (14.3%)	2 (4.3%)	
**Child-Pugh classification**				1.000
A	79 (89.8%)	38 (90.5%)	41 (89.1%)	
B	9 (10.2%)	4 (9.5%)	5 (10.9%)	
**Tumor diameter (cm)**				0.328
< 5 cm	63 (71.6%)	28 (66.7%)	35 (76.1%)	
≥ 5 cm	25 (28.4%)	14 (33.3%)	11 (23.9%)	
**No. of tumors**				0.327
< 3	59 (67.0%)	26 (61.9%)	33 (71.7%)	
≥ 3	29 (33.0%)	16 (38.1%)	13 (28.3%)	
**Extrahepatic metastasis**				0.659
Yes	63 (71.6%)	31 (73.8%)	32 (69.6%)	
No	25 (28.4%)	11 (26.2%)	14 (30.4%)	
**Portal vein** **tumor thrombus**				0.742
No	78 (88.6%)	38 (90.5%)	40 (87.0%)	
Yes	10 (11.4%)	4 (9.5%)	6 (13.0%)	
**AFP level (ng/mL)**				0.904
≥ 400	32 (36.4%)	15 (35.7%)	17 (37.0%)	
< 400	56 (63.6%)	27 (64.3%)	29 (63.0%)	
**MVI**				0.808
Positive	47 (53.4%)	23 (54.8%)	24 (52.2%)	
Negative	41 (46.6%)	19 (45.2%)	22 (47.8%)	
**Interval of recurrence from initial treatment**				0.619
≥ 1 year	23 (26.1%)	12 (28.6%)	11 (23.9%)	
<1 year	65 (73.9%)	30 (71.4%)	35 (76.1%)	
**ALT level (U/L)**	28.0 (19.0, 41.0)	27.0 (18.0, 37.0)	29.0 (19.0, 56.0)	0.268
**AST level (U/L)**	30.0 (19.0, 45.0)	29.0 (20.0, 38.0)	32.0 (19.0, 55.0)	0.408
**GGT level (U/L)**	60.0 (37.0, 110.0)	62.0 (38.0, 105.0)	58.0 (36.0, 118.0)	0.593
**Total bilirubin level (mg/dL)**	14.0 (11.0, 19.0)	14.0 (10.0, 20.0)	14.0 (11.0, 19.0)	0.631
**ALB level (g/L)**	41.1 (38.4, 45.0)	41.2 (38.5, 43.4)	41.0 (37.8, 45.3)	0.977
**Platelet count**	120.0 ± 58.0	127.0 ± 60.0	113.0 ± 55.0	0.271
**Lymphocyte count**	1.22 (0.88, 1.58)	1.32 (0.93, 1.87)	1.13 (0.86, 1.44)	0.134
**Neutrophil count**	2.67 (1.85, 3.29)	2.94 (2.09, 3.32)	2.44 (1.65, 3.28)	0.333

AFP, a-fetoprotein; ALB, albumin; ALT, alanine amiotransferase; AST, aspartate transaminase; GGT, g-glutamyl transpeptidase; MVI, microvascular invasion; No. of tumors, number of tumors.

### Survival analysis

**Fig. 2:**
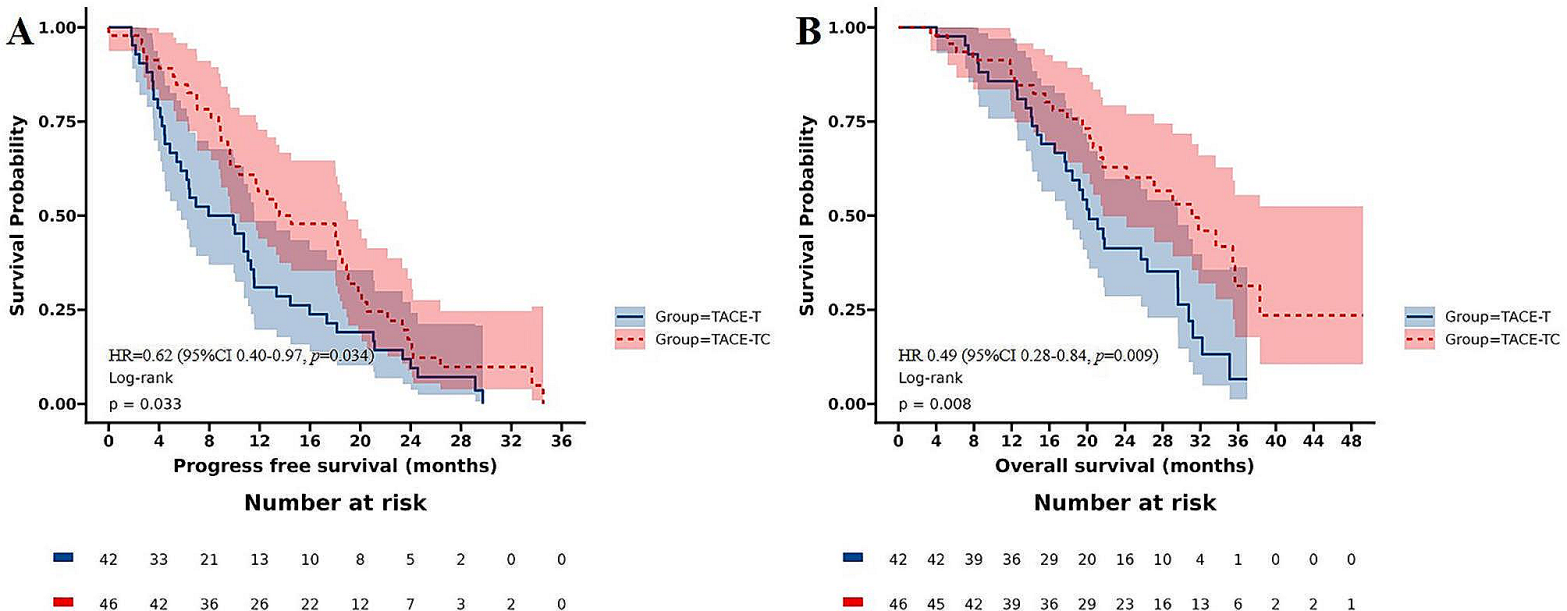
OS and PFS of patients receiving the different treatments. (**A**) OS of all patients. (**B**) PFS of all patients

The median PFS in TACE-TC group was significantly longer than that in TACE-T group [14.0(95%CI 10.4 ∼ 19.0) months vs. 8.9(95%CI 5.7 ∼ 11.6) months; Fig. 2A], and the difference was statistically significant (HR = 0.62, 95%CI 0.40 ∼ 0.97; *p* = 0.034). Similarly, the median OS in TACE-TC group was longer than in TACE-T group [31.1(95% CI 21.7 ∼ NA) months vs. 20.2(95%CI 17.8 ∼ 29.6) months; Fig. 2B] and the difference was statistically significant (HR 0.49,95%CI 0.28–0.84; *p* = 0.009).

### Tumor and laboratory response

**Fig. 3:**
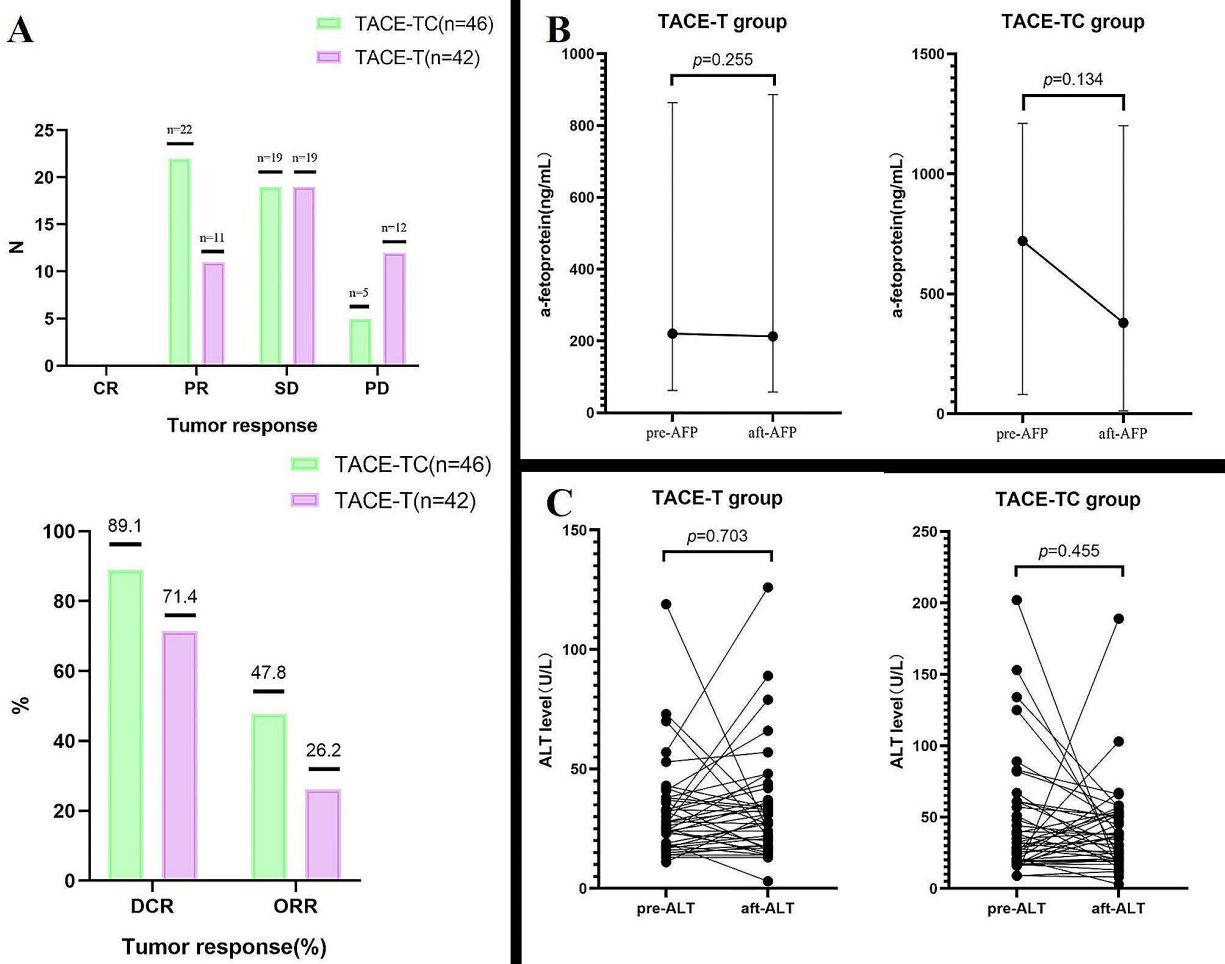
Laboratory changes and treatment response. (**A**) tumor responses in each cohort; (**B**) Median change AFP from Before and after treatment in patients with baseline AFP > 7 ng/mL (*n* = 62); (**C**) Changes in liver function indicators of each patient before and after treatment. CR, complete response; PD, progressive disease; PR, partial response; SD, stable disease; DCR (CR + PR + SD), disease control rate; ORR (CR + PR), objective response rate; AFP, a-fetoprotein; ALT, alanine aminotransferase

The ORR and DCR in the TACE-TC group were significantly higher than that in the TACE-T groups (ORR: 47.8% vs. 26.2%, *p* = 0.036; DCR: 89.1% vs. 71.4%, *p* = 0.036) (Fig. 3A). After three months of treatment, patients (*n* = 62) with baseline AFP greater than 7 ng/mL had varying degrees of changes in AFP levels. The median baseline AFP in the TACE-T group was 220.5 ng/mL (IQR 62.4, 864.0), and the median AFP after treatment was 212.8 ng/mL (IQR 57.8, 886.8). The median baseline AFP in the TACE-TC group was 720.2 ng/mL (IQR 79.8, 1210.0), and the median post-treatment AFP was 379.5 ng/mL (IQR 12.1, 1201.0). Compared with the TACE-T group, the AFP level in the TACE-TC group decreased more significantly after 3 months of treatment (Fig. 3B). As for the changes in ALT, both groups were within the controllable range (Fig. 3C). The median baseline ALT in the TACE-T group was 26.5 U/L (IQR 18.0, 37.3), and the median ALT after treatment was 27.5 U/L (IQR 17.8, 36.3); the median baseline ALT in the TACE-TC group was 28.5 U/L (IQR 18.8, 58.0), and after treatment Median ALT was 36.0 U/L (IQR 20.0, 51.3).

### Prognostic factor analysis for PFS and OS

Based on univariate analyses, the OS has been significantly associated with child–Pugh classification, portal vein tumor thrombus, AFP, treatment option, and interval of recurrence from initial treatment in this study (*p* < 0.01). Upon multivariate analysis treatment option (HR = 2.013, 95%CI 1.162 ∼ 3.489, *p* = 0.013) was independent prognostic factors of OS (Table 2). Univariate analysis revealed that portal vein tumor thrombus, treatment option, and interval of recurrence from initial treatment were factors associated with PFS (*p* < 0.01). Multivariate analysis indicated that portal vein tumor thrombus (HR = 2.103, 95%CI 1.072 ∼ 4.125, *p* = 0.031), treatment option (HR = 1.621, 95%CI 1.045 ∼ 2.514, *p* = 0.031) and interval of recurrence from initial treatment (HR = 0.494, 95%CI 0.289 ∼ 0.844, *p* = 0.010) were independent prognostic factors of PFS (Table 3).

**Table 2:** Univariate and multivariate Cox regression analyses for OS.

Characteristic	Univariate analysis			Multivariate analysis		
	HR	95%CI	P	HR	95%CI	P
Child–Pugh classification(B/A)	0.446	0.210 ∼ 0.951	0.037	0.658	0.289 ∼ 1.499	0.319
No. of tumors (≥ 3/<3)	1.191	0.671 ∼ 2.114	0.550			
Tumor diameter (≥ 5 cm/ <5 cm)	0.873	0.489 ∼ 1.561	0.648			
Portal vein tumor thrombus (yes/no)	2.881	1.389 ∼ 5.975	0.004	1.913	0.868 ∼ 4.214	0.107
Extrahepatic metastases (yes/no)	1.203	0.645 ∼ 2.244	0.562			
AFP (≥ 400ng/mL/ < 400ng/mL)	1.914	1.132 ∼ 3.234	0.015	1.608	0.929 ∼ 2.782	0.089
Treatment option (TACE + T/TACE + TC)	2.056	1.194 ∼ 3.540	0.009	2.013	1.162 ∼ 3.489	0.013
Interval of recurrence from initial treatment(<1 year/≥1 year)	0.473	0.248 ∼ 0.900	0.023	0.534	0.277 ∼ 1.030	0.061
MVI(Positive/Negative)	1.109	0.658 ∼ 1.869	0.699			

AFP, a-fetoprotein; MVI, microvascular invasion; No. of tumors, number of tumors.

**Table 3:** Univariate and multivariate Cox regression analyses for PFS.

Characteristic	Univariate analysis			Multivariate analysis		
	HR	95%CI	P	HR	95%CI	P
Child–Pugh classification(B/A)	1.153	0.548 ∼ 2.426	0.708			
No. of tumors (≥ 3/<3)	1.291	0.806 ∼ 2.067	0.288			
Tumor diameter (≥ 5 cm/<5 cm)	1.092	0.673 ∼ 1.771	0.723			
Portal vein tumor thrombus (yes/no)	1.928	0.991 ∼ 3.751	0.053	2.103	1.072 ∼ 4.125	0.031
Extrahepatic metastases(yes/no)	1.034	0.633 ∼ 1.689	0.893			
AFP (≥ 400ng/mL/<400ng/mL)	1.299	0.831 ∼ 2.032	0.251			
Treatment option (TACE + T/TACE + TC)	1.605	1.035 ∼ 2.488	0.034	1.621	1.045 ∼ 2.514	0.031
Interval of recurrence from initial treatment(<1 year/≥1 year)	0.519	0.305 ∼ 0.881	0.015	0.494	0.289 ∼ 0.844	0.010
MVI(Positive/Negative)	1.298	0.838 ∼ 2.008	0.242			

AFP, a-fetoprotein; MVI, microvascular invasion; No. of tumors, number of tumors.

### Subgroup Analysis

**Fig. 4:**
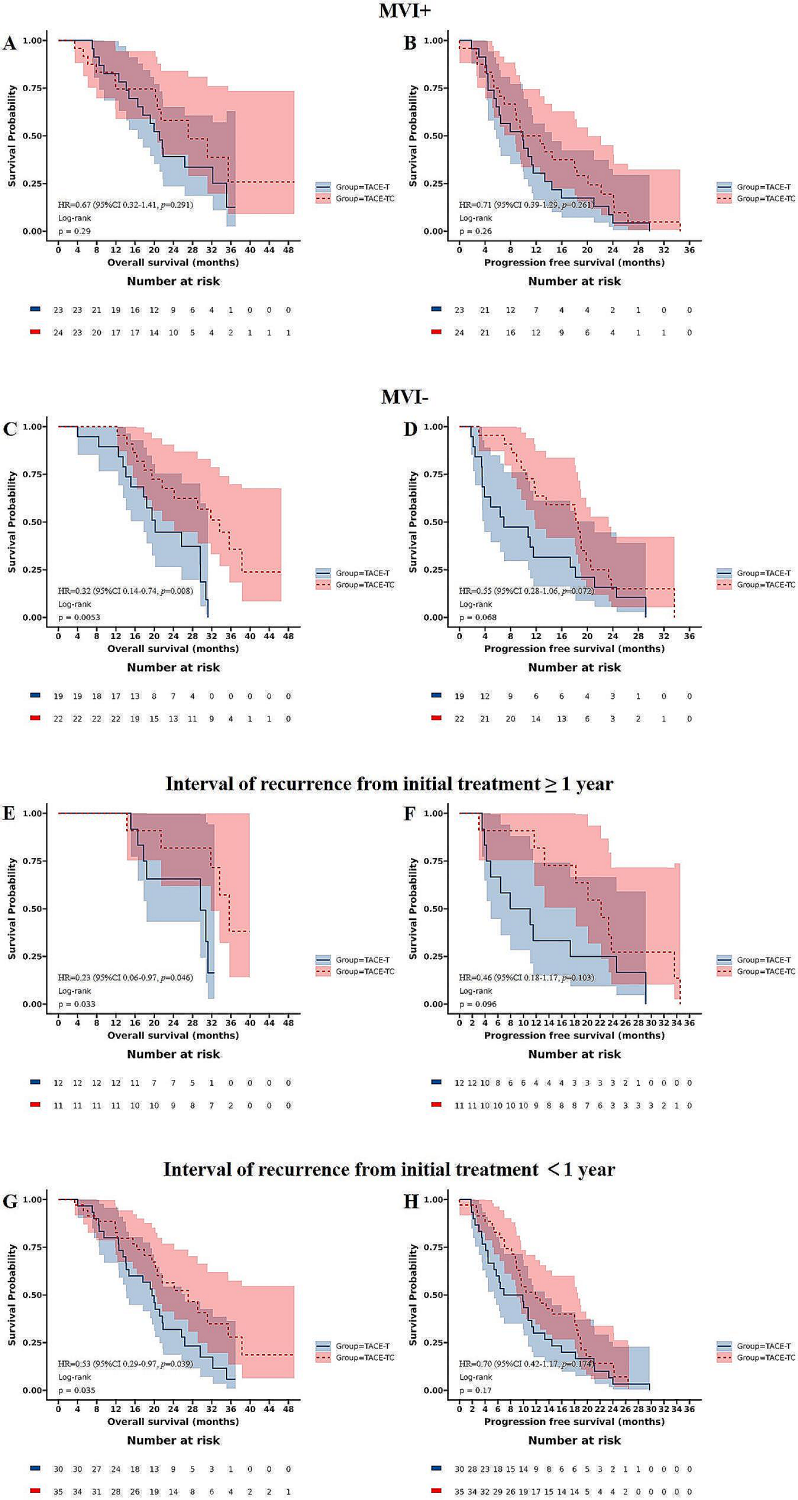
PFS and OS of patients receiving the different treatments. (**A**) OS of patients with MVI positive. (**B**) OS of patients with MVI positive; (**C**)OS of patients with MVI negative; (**D**) PFS of patients with MVI negative; (**E**) OS of patients with interval of recurrence from initial treatment ≥ 1 year; (**F**) PFS of patients with interval of recurrence from initial treatment ≥ 1 year; (G)OS of patients with interval of recurrence from initial treatment <1 year; (H) PFS of patients with interval of recurrence from initial treatment <1 year. MVI, microvascular invasion

Among patients with MVI positive, the median OS was 27.1 months (95%CI 20.6 ∼ NA) in the TACE-TC group and 21.1 months (95%CI 16.6 ∼ NA) in the TACE-T group (Fig. 4A, *p* = 0.290). The corresponding PFS was 11.1 months (95%CI 8.7 ∼ 20.1) in the TACE-TC group and 9.9 months (95%CI 5.7 ∼ 14.4) in the TACE-T group (Fig. 4B, *p* = 0.260). In patients with MVI negative, the median OS was 33.6 months (95%CI 24.2 ∼ NA) in the TACE-TC group and 20.2 months (95%CI 17.8 ∼ NA) in the TACE-T group (Fig. 4C, *p* = 0.005). The corresponding PFS was 18.4 months (95% CI 11.9 ∼ 23.3) in the TACE-TC group and 6.9 months (95%CI 3.9 ∼ 21.1) in the TACE-T group (Fig. 4D, *p* = 0.068). In patients with interval of recurrence from initial treatment ≥ 1 year, the median OS was 35.7 months (95%CI 31.8 ∼ NA) in the TACE-TC group and 29.6 months (95%CI 18.4 ∼ NA) in the TACE-T group (Fig. 4E, *p* = 0.033). The corresponding PFS was 22.2 months (95%CI 18.2 ∼ NA) in the TACE-TC group and 9.5 months (95%CI 4.9 ∼ NA) in the TACE-T group (Fig. 4F, *p* = 0.096).In patients with interval of recurrence from initial treatment<1 year, the median OS was 27.1 months (95%CI 20.3 ∼ NA) in the TACE-TC group and 19.5 months (95%CI 14.2 ∼ 26.4) in the TACE-T group (Fig. 4G, *p* = 0.035). The corresponding PFS was 11.9 months (95%CI 8.9 ∼ 18.6) in the TACE-TC group and 8.4 months (95%CI 5.4 ∼ 13.3) in the TACE-T group (Fig. 4H, *p* = 0.170).

### Safety

No treatment-related death occurred in the two groups. In the TACE-TC group, the most common adverse reaction was elevated bilirubin, neutropaenia, and elevated AST. The most common adverse reactions in the TACE-T group were elevated bilirubin, elevated AST, and thrombocytopaenia. There was no significant difference in the incidence and severity of adverse reactions between the two groups (*p* < 0.05, Table 4). 1 patient in the TACE-T group stopped the drug for a short period due to adverse reactions and resumed normal treatment after symptomatic treatment, and 3 patients in the TACE-TC group stopped the drug for a short period due to intolerance of adverse reactions.

**Table 4:** Treatment-related adverse events

n(%)	All grade			Grade 3–4		
	TACE-TC(*n* = 46)	TACE-T(*n* = 42)	P	TACE-TC(*n* = 46)	TACE-T(*n* = 42)	P
Hypertension	10(21.7)	13(31.0)	0.326	2(4.3)	4(9.5)	0.419
Diarrhea	8(17.4)	7(16.7)	0.928	1(2.1)	2(4.8)	0.604
Appetite decreased	7(15.2)	5(11.9)	0.651	0	0	-
Fatigue	5(10.9)	3(7.1)	0.716	0	0	-
RCCEP	11(23.9)	0	0.001	0	0	-
Hypothyroidism	4(8.6)	0	0.118	0	0	-
Hoarseness	0	1(2.4)	0.477	0	0	-
Hand–foot skin reaction	4(8.6)	9(21.4)	0.133	1(2.1)	2(4.8)	0.604
Hemorrhagic cystitis	1(2.2)	0	1.000	0	0	-
Thrombocytopaenia	12(26.1)	14(33.3)	0.457	3(6.5)	2(4.8)	1.000
Neutropaenia	14(30.4)	10(23.8)	0.486	0	1(2.4)	0.477
Decreased WBC count	11(23.9)	9(21.4)	0.781	1(2.1)	0	1.000
Lymphocytopenia	12(26.1)	10(23.8)	0.805	2(4.3)	0	0.495
Elevated AST	19(41.3)	20(47.6)	0.551	4(8.7)	5(11.9)	0.731
Elevated ALT	14(30.4)	13(31.0)	0.958	2(4.3)	0	0.495
Elevated bilirubin	21(45.7)	18(42.9)	0.792	0	3(7.1)	0.105

RCCEP, Reactive cutaneous capillary endothelial proliferation; WBC, white blood cell; ALT, alanine amiotransferase; AST, aspartate transaminase.

## Discussion

There is no clear consensus on the standard salvage treatment for RHCC, and treatment methods including surgery, interventional therapy, radiotherapy, and drug therapy have achieved certain results in RHCC [29–31]. TACE is the most widely used treatment for RHCC [32]. How to further improve the efficacy of TACE, especially for those patients with vascular invasion or extrahepatic metastasis, is the key to the long-term benefit of patients with RHCC.

Given the pattern of TACE combined with systemic therapy in primary liver cancer, several studies have reported the efficacy of combination therapy in RHCC. Peng et al. [33]. reported that the mOS and median time to progression (mTTP) of TACE and radiofrequency ablation combined with sorafenib in the treatment of RHCC were longer than those of sorafenib alone (mOS: 14.0 months vs. 9.0 months, *p* < 0.001; mTTP: 7.0 months vs. 4.0 months, *p* < 0.001). The research results of Li et al. [34]. showed that MTT combined with PD-1 therapy in RHCC patients had a survival advantage compared with patients receiving MTT therapy alone, and the median OS was prolonged by 17.8 months. Wang et al. [22]. conducted the first study on the safety and efficacy of TACE combined with MTT and PD-1 inhibitors in the treatment of RHCC. The results of the study showed that compared with TACE single drug or TACE-lenvatinib, TACE-lenvatinib-PD-1 inhibitors can improve the survival rate of RHCC, but more toripalimab (30/54, 55.6%) and sintilimab (21/54, 38.8%) were used in this study, camrelizumab was only used in 3 (3/54, 5.6%) patients. The occurrence of liver cancer in China is closely related to chronic infection of hepatitis B virus [35], T-cell dysfunction in hepatitis B patients leaves the tumor microenvironment in an immunosuppressed state. Previous research shows the TACE process can improve this situation by promoting an inflammatory environment that promotes the activity of T cells [36] and facilitate the anti-tumor effect of ICI [37]. Camrelizumab was approved for use based on the results of a national multi-center phase II clinical study of advanced hepatocellular carcinoma in China that failed previous systemic treatment [23]. Whether as monotherapy or combination regimen, camrelizumab has shown good efficacy in primary liver cancer. We speculate that camrelizumab may be particularly beneficial in patients with HBV-related HCC. Therefore, to understand the safety and efficacy of TACE-T combined with camrelizumab in the treatment of RHCC, we conducted this study and compared them with those of TACE combined with MTT.

Our study shows that in unresectable RHCC, TACE-TC was more effective than TACE-T. Patients treated with TACE-T showed a median OS of 20.2 months, a median PFS of 8.9 months, and an DCR of 71.4% according to mRECIST criteria. The mOS, mPFS, and DCR of the TACE-TC group were higher than those of the TACE-T group. The disease burden of the patient is strongly correlated with the serum level of alpha-fetoprotein produced by the tumor, making it a reliable tumor marker [38]. The fluctuations in alpha-fetoprotein levels before and after treatment also indicate the effectiveness of the treatment [39]. This study observed a more significant decrease in alpha-fetoprotein levels in the TACE-TC group compared to the TACE-T group. This shows that TACE-TC is better than TACE-T in inhibiting tumor activity, which is consistent with the comparison of DCR and ORR. This result can be attributed to the synergistic effect of camrelizumab, MTT, and TACE. Tumor necrosis after TACE can induce immune activation and upregulate the expression of PD-1 [18, 40], and anti-angiogenic therapy can reduce the immunosuppression of the tumor immune microenvironment and promote the infiltration of tumor T cells [41]. With the addition of camrelizumab, a chain reaction is triggered to improve the anti-tumor efficacy.

According to earlier research, relapse tumors differ from primary tumors in having fewer regulatory T cells, more dendritic cells, and more infiltrating CD8 T cells [42]. The enrichment of these cells was associated with a worse prognosis. This may be a potential immune evasion mechanism of RHCC. At present, there are few clinical studies on immunotherapy for RHCC. Guo et al. [43] investigated the effectiveness of TACE combined with camrelizumab in treating RHCC. Although the study has not yet reached the median OS, the median PFS and ORR showed no significant difference between TACE combined with camrelizumab and TACE alone (mPFS 9 months vs. 6 months, *p* = 0.586; ORR 40% vs. 56.9%, *p* = 0.201). However, in two previous studies on RHCC, combination therapy based on PD-1 inhibitors was significantly superior to MTT alone or TACE combined with MTT [22, 34]. In the results of this study, the treatment mode of TACE and MTT combined with camrelizumab improved the treatment outcome of RHCC patients, and the results of this study can be used as a reference for other studies to further explore the application of immunotherapy in RHCC.

In the subgroup analysis of this study, it was found that the OS of the TACE-TC group was better than that of TACE-T, whether in the subgroup with interval of recurrence from initial treatment <1 year or in the subgroup with interval of recurrence from initial treatment ≥ 1 year. Therefore, for advanced RHCC, early combination immunotherapy is beneficial to the long-term survival of patients.

The TACE-TC group had immune-related adverse events due to the addition of camrelizumab, and these adverse reactions were relieved after the short-term suspension of camrelizumab or topical glucocorticoids. The incidence and severity of AEs were also comparable between the TACE-TC and TACE-T groups. These findings suggested that TACE-TC and TACE-T therapies were both tolerable, and the addition of a camrelizumab to TACE-T did not significantly enhance the risk of adverse events compared to TACE-T. This showed that TACE-TC had an acceptable safety profile.

There are several limitations to this study. First, this study was conducted retrospectively and despite the involvement of a multidisciplinary team in developing treatment plans, bias was unavoidable due to the selection of patients. Second, our study was conducted at a single center. Therefore, it is necessary to conduct prospective multicenter clinical trials to verify our findings in the future. Notably, all the molecular targeted drugs and camrelizumab applied in the present study are recommended for HCC in treatment guidelines. We used three molecular targeted drugs because they have similar targets inspired by the new clinical research strategy perspective of Menis et al. [44]. This design can validate a treatment strategy involving a mixture of agents and has been successfully conducted in several trials [45, 46].

## Conclusion

In conclusion, the safety of TACE-TC and TACE-T in the treatment of RHCC is manageable. TACE-TC treatment has shown better response and improved survival outcomes compared to TACE-T for RHCC.

## Data Availability

“The datasets generated and/or analyzed during the current study are available from the corresponding author on reasonable request.”

## Abbreviations

AEs: Adverse events
ALB: Albumin
AFP: a-Fetoprotein
ALT: Alanine aminotransferase
AST: Aspartate aminotransferase
CR: Complete response
DCR: Disease control rate
HCC: hepatocellular carcinoma
ICIs: Immune checkpoint inhibitors
MTT: Molecular targeted therapies
MVI: Microvascular invasion
ORR: Objective response rate
OS: Overall survival
PD-1: Programmed cell death protein-1
PR: Partial response
PD: Progressive disease
PFS: Progression-free survival
PVTT: Portal vein tumor thrombus
RHCC: Recurrent hepatocellular carcinoma
RCCEP: Reactive cutaneous capillary endothelial proliferation
SD: Stable disease
TACE: Transarterial Chemoembolization
VEGF: Vascular endothelial growth factor
WBC: White blood cell
